# Serum Concentrations of Angiopoietin-2 and Soluble fms-Like Tyrosine Kinase 1 (sFlt-1) Are Associated with Coagulopathy among Patients with Acute Pancreatitis

**DOI:** 10.3390/ijms18040753

**Published:** 2017-04-02

**Authors:** Paulina Dumnicka, Beata Kuśnierz-Cabala, Mateusz Sporek, Małgorzata Mazur-Laskowska, Krzysztof Gil, Marek Kuźniewski, Piotr Ceranowicz, Zygmunt Warzecha, Artur Dembiński, Joanna Bonior, Ryszard Drożdż

**Affiliations:** 1 Department of Medical Diagnostics, Jagiellonian University Medical College, Medyczna 9, 30-688 Kraków, Poland; paulina.dumnicka@uj.edu.pl (P.D.); ryszard.drozdz@uj.edu.pl (R.D.); 2 Department of Diagnostics, Chair of Clinical Biochemistry, Jagiellonian University Medical College, Kopernika 15A, 31-501 Kraków, Poland; 3 Department of Anatomy, Jagiellonian University Medical College, Kopernika 12, 31-034 Kraków, Poland; msporek1983@gmail.com; 4 Surgery Department, The District Hospital, Szpitalna 22, 34-200 Sucha Beskidzka, Poland; 5 Department of Diagnostics, University Hospital, Kopernika 15B, 31-501 Kraków, Poland; mbmazur@cyf-kr.edu.pl; 6 Department of Pathophysiology, Jagiellonian University Medical College, Czysta 18, 31-121 Kraków, Poland; mpgil@cyf-kr.edu.pl; 7 Chair and Department of Nephrology, Jagiellonian University Medical College, Kopernika 15, 31-501 Kraków, Poland; marek.kuzniewski@uj.edu.pl; 8 Department of Physiology, Jagiellonian University Medical College, Grzegórzecka 16, 31-531 Kraków, Poland; piotr.ceranowicz@uj.edu.pl (P.C.); zygmunt.warzecha@uj.edu.pl (Z.W.); artur.dembinski@uj.edu.pl (A.D.); 9 Department of Medical Physiology, Jagiellonian University Medical College, Michałowskiego 12, 31-126 Kraków, Poland; joanna.bonior@uj.edu.pl

**Keywords:** acute pancreatitis, disseminated intravascular coagulation, D-dimer, angiopoietin-2, soluble fms-like tyrosine kinase 1

## Abstract

In severe acute pancreatitis (SAP), systemic inflammation leads to endothelial dysfunction and activation of coagulation. Thrombotic disorders in acute pancreatitis (AP) include disseminated intravascular coagulation (DIC). Recently, angiopoietin-2 and soluble fms-like tyrosine kinase 1 (sFlt-1) were proposed as markers of endothelial dysfunction in acute states. Our aim was to assess the frequency of coagulation abnormalities in the early phase of AP and evaluate the relationships between serum angiopoietin-2 and sFlt-1 and severity of coagulopathy. Sixty-nine adult patients with AP were recruited: five with SAP, 15 with moderately severe AP (MSAP) and 49 with mild AP. Six patients were diagnosed with DIC according to International Society on Thrombosis and Haemostasis (ISTH) score. All patients had at least one abnormal result of routine tests of hemostasis (low platelet count, prolonged clotting times, decreased fibrinogen, and increased D-dimer). The severity of coagulopathy correlated with AP severity according to 2012 Atlanta criteria, bedside index of severity in AP and duration of hospital stay. D-dimers correlated independently with C-reactive protein and studied markers of endothelial dysfunction. Angiopoietin-2, D-dimer, and ISTH score were best predictors of SAP, while sFlt-1 was good predictor of MSAP plus SAP. In clinical practice, routine tests of hemostasis may assist prognosis of AP.

## 1. Introduction

Acute pancreatitis (AP) is an inflammatory disorder, characterized by a spectrum of severity, ranging from mild disease in most patients, to severe life-threatening condition [1,2]. Current guidelines [3] classify the disease course as mild (MAP), moderately-severe (MSAP) and severe AP (SAP) on the basis of organ failure (transient or persistent), and local and systemic complications. Organ failure (including cardiovascular, pulmonary and renal) occurring in the early phase of AP is the most important determinant of severity and the main cause of early deaths [3]. Severe course of AP is associated with excessive systemic inflammation, involving systemic activation and dysfunction of endothelial cells, leading to vascular leak syndrome and organ failure [4].

Angiopoietin-2 and soluble fms-like tyrosine kinase 1 (sFlt-1) have been proposed as novel predictors of severity in acute states, such as sepsis or AP [5,6,7,8,9,10,11]. Angiopoietin-2 is an angiogenic growth factor, binding and inhibiting Tie-2 receptor on endothelial cells, which has been associated with destabilization of endothelium and increased vascular leakage [12,13]. Flt-1 is a receptor for vascular endothelial growth factor and placental growth factor, its soluble form (sFlt-1) acts as a decoy receptor [14]. Both angiopoietin-2 and sFlt-1 may be considered markers of endothelial dysfunction in acute states. We have previously reported the associations between serum concentrations of angiopoietin-2 and the development of acute kidney injury and renal failure in the course of AP as well as the severity of AP [7]. In addition, we have reported the association between serum sFlt-1 concentrations in the early phase of AP and the severity of the disease [8].

Endothelial dysfunction may result in activation of platelets and coagulation. Clinically significant hemorrhagic and thrombotic disorders were observed, respectively, among 6% and 7% of patients who died due to AP [15]. Several coagulopathies were reported as the complications of AP, including among others disseminated intravascular coagulation (DIC) and thrombotic thrombocytopenic purpura [16,17,18,19]. The results of laboratory tests used to assess hemostasis were shown to predict severity of AP and related mortality with reasonable diagnostic utility, in some reports exceeding those observed for C-reactive protein [16,20]. In particular, high diagnostic accuracy to predict SAP was reported for D-dimer plasma concentrations [16,20,21,22,23].

The aim of the study was to assess the frequency of coagulopathy in a cohort of consecutive patients with AP in the early phase of the disease. Moreover, we studied the associations between the serum concentrations of angiopoietin-2 and sFlt-1 and the presence as well as the severity of abnormalities of coagulation in the early phase of AP.

## 2. Results

Sixty-nine patients (35 men and 34 women; mean age 69 ± 18) were included in the study, among them 49 were diagnosed with MAP, 15 with MSAP, and 5 with SAP.

During the first 48 h of AP, six patients (7% of the total cohort) were assigned six points or more in the International Society on Thrombosis and Haemostasis (ISTH) score for overt DIC [24], thus fulfilling the criteria of overt DIC. The diagnosis of DIC was significantly associated with more severe AP, higher bedside index of severity in AP (BISAP) [25] and Glasgow [26] severity scores, longer hospital stays, and higher mortality (Table 1). Patients subsequently diagnosed with DIC had longer prothrombin times and higher plasma concentrations of D-dimer at admission, as well as higher serum urea (Table 1).

**Figure 1:**
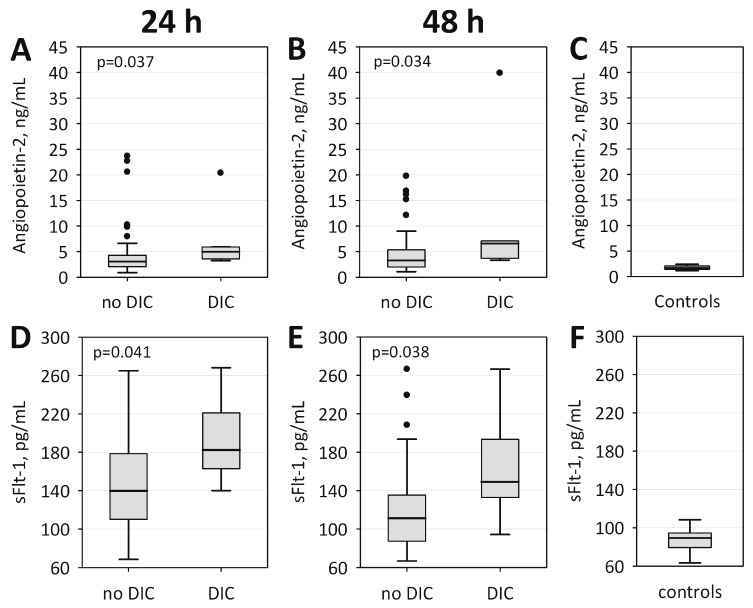
Serum angiopoietin-2 and sFlt-1 concentrations on: admission (**A**,**D**); and on Day 2 of hospital stay (**B**,**E**), among acute pancreatitis (AP) patients with ISTH score for overt DIC below 5 (no DIC) and ≥5 points (DIC). Serum: angiopoietin-2 (**C**); and sFlt-1 (**F**) concentrations observed among 21 healthy controls are presented for comparison. Data are shown as median, interquartile range (box), non-outlier range (whiskers), and outliers (points). Abbreviations: sFlt-1, soluble fms-like tyrosine kinase 1; DIC, diffuse intravascular coagulation; ISTH, International Society on Thrombosis and Haemostasis.

Twenty-one healthy volunteers (12 women and nine men; mean age 51 ± 10 years) provided serum samples in order to compare angiopoietin-2 and sFlt-1 concentrations between healthy controls and AP patients. In the control group, angiopoietin-2 concentrations were in the range of 1.17–2.41 ng/mL with median (lower-upper quartile) of 1.69 (1.44–2.08) ng/mL and sFlt-1 concentrations were in the range of 63–108 pg/mL with median (lower-upper quartile) of 89 (79–94) pg/mL. Both angiopoietin-2 (*p <* 0.001 at 24 h and *p <* 0.001 at 48 h) and sFlt-1 (*p <* 0.001 at 24 h and *p <* 0.001 at 48 h) were significantly higher in AP patients than in controls (Figure 1). In multiple logistic regression, these differences between AP patients and controls were independent of age. The concentrations of angiopoietin-2 and sFlt-1 were also significantly higher among AP patients with DIC comparing to patients without DIC, both on admission and on the second day of hospital stay (Table 1, Figure 1).

**Figure 2:**
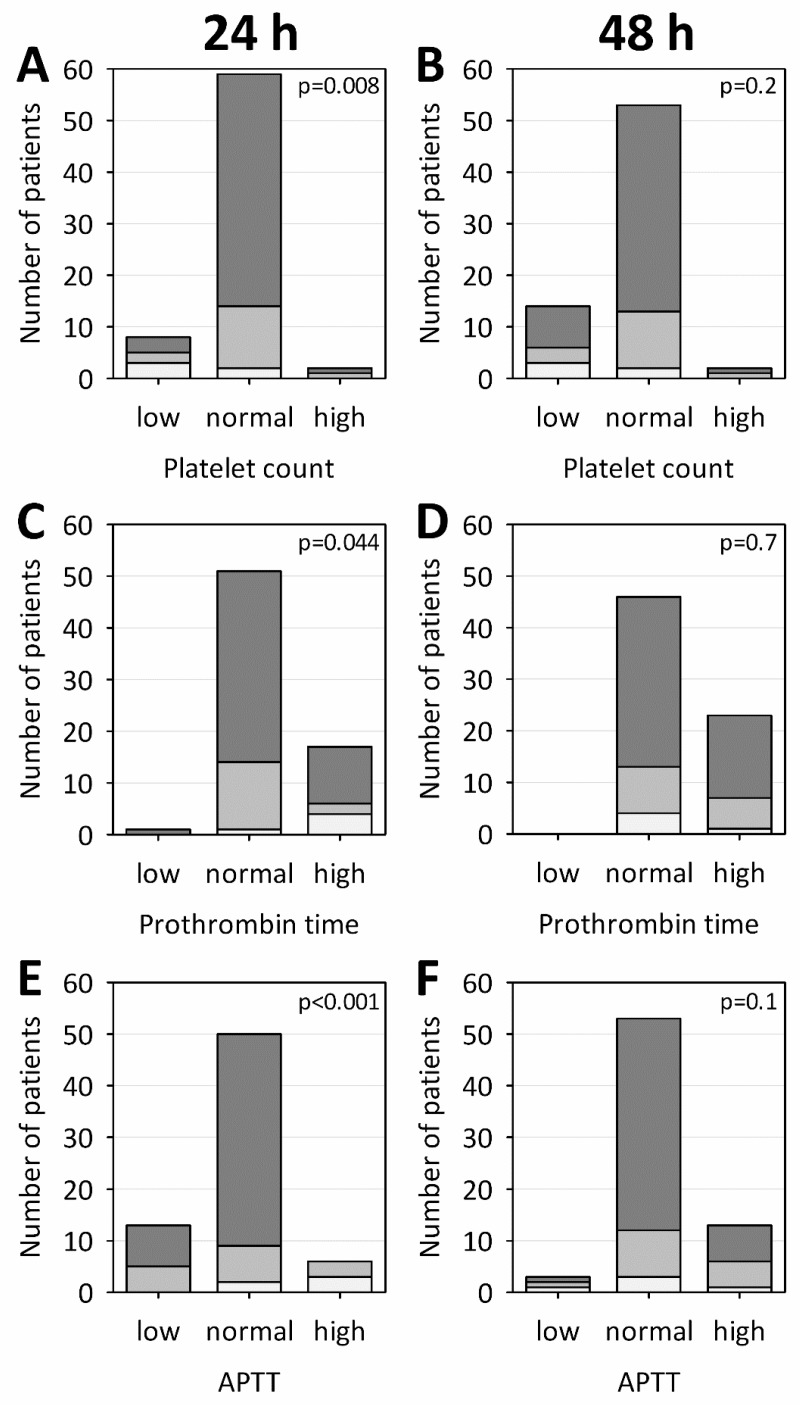
Numbers of patients with platelet counts (**A**,**B**); prothrombin times (**C**,**D**); and activated partial thromboplastin times (APTT) (**E**,**F**) below (low), within (normal) and above (high) the reference intervals. Panels **A**, **C** and **E** show the results obtained at 24 h and Panels **B**, **D** and **F** at 48 h from the onset of AP symptoms. Numbers of patients with mild AP (MAP), moderately severe AP (MSAP) and severe AP (SAP) are represented by dark, medium and light boxes, respectively; p-values for the chi-squared tests are shown on the graphs.

**Figure 3:**
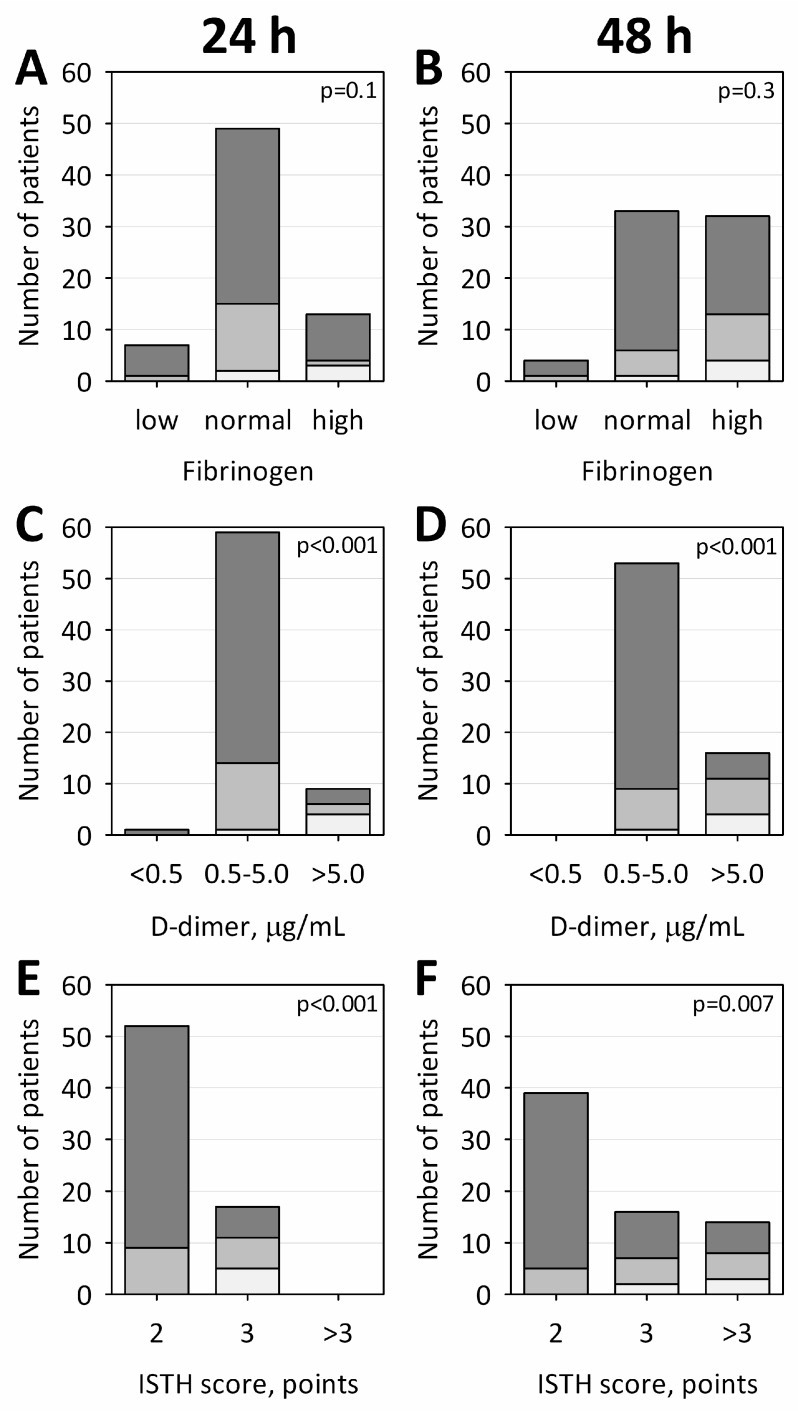
Numbers of patients with fibrinogen concentrations below (low), within (normal) and above (high) the reference interval (**A**,**B**); with D-dimer concentrations below reference limit (<0.5 µg/mL), increased up to 10 times (0.5–5.0 µg/mL) and increased more than 10 times (> 5. 0 µg/mL) (**C**,**D**); and with ISTH score for overt DIC of 2, 3, and more than 3 points (**E**,**F**). Panels **A**, **C** and **E** show the results obtained at 24 h and Panels **B**, **D** and **F** at 48 h from the onset of AP symptoms. Numbers of patients with MAP, MSAP and SAP are represented by dark, medium and light boxes, respectively; *p*-values for the chi-squared tests are shown on the graphs.

The abnormal results of routine tests of hemostasis, suggestive of consumptive coagulopathy, were significantly associated with AP severity. On admission, low platelet counts were observed in 60% of patients with SAP, 13% with MSAP and 6% with MAP; prothrombin time was prolonged in 80% of patients with SAP, 13% with MSAP and 22% with MAP; and activated partial thromboplastin time (APTT) was prolonged in 60% of those with SAP, 20% with MSAP and none patients with MAP (Figure 2A,C,E, respectively). Following treatment, these differences became non-significant already on Day 2 of hospital stay (Figure 2B,D,F). No significant associations were detected between AP severity and fibrinogen concentrations, however, patients with SAP tended towards high concentrations (Figure 3A,B). High concentrations of D-dimer were associated with more severe AP both at 24 and 48 h after the onset of AP symptoms. Severely increased D-dimer levels (>5 µg/mL) were observed in 80% of patients with SAP, 13% of those with MSAP and 6% with MAP on Day 1, and 80% of patients with SAP, 47% with MSAP, and 10% with MAP on Day 2, respectively (Figure 2C,D). Consequently, significantly higher results in ISTH score for overt DIC were observed in SAP and MSAP patients versus those with MAP (Figure 2E,F). On Day 2 of AP, 40% of patients with SAP, 20% of those with MSAP and 2% with MAP were assigned ≥5 points in ISTH score, supporting the diagnosis of overt DIC. Consistent with the fact that D-dimer concentrations were increased above the reference limit (0.5 µg/mL) in all the studied patients (except for one lower result achieved on admission), the lowest ISTH score among studied patients was 2 points.

Positive correlations were observed between log-transformed C-reactive protein and fibrinogen concentrations (*R =* 0.54; *p <* 0.001 on Day 1 and *R =* 0.27; *p =* 0.025 on Day 2), D-dimer (*R =* 0.45; *p <* 0.001 on Day 1 and *R =* 0.64; *p <* 0.001 on Day 2), APTT (*R =* 0.33; *p =* 0.007 on Day 1), and ISTH score (*R =* 0.29; *p =* 0.017 on Day 1 and *R =* 0.49; *p <* 0.001 on Day 2). Only fibrinogen concentrations correlated with amylase activity (for log-transformed variables: *R =* −0.32; *p =* 0.010 on Day 1). On admission, log-transformed D-dimer (*R =* 0.51; *p <* 0.001), APTT (*R =* 0.34; *p =* 0.005), platelet count (*R =* −0.29; *p =* 0.017) and ISTH score (*R =* 0.43; *p <* 0.001) significantly correlated with BISAP score, and log (D-dimer) (*R =* 0.34; *p =* 0.004), platelet count (*R =* −0.33; *p =* 0.006) and ISTH score (*R =* 0.24; *p =* 0.049) significantly correlated with Glasgow score. Moreover, log (fibrinogen) (*R =* 0.27; *p =* 0.028 on admission), log (D-dimer) (*R =* 0.46; *p <* 0.001 on Day 1 and *R =* 0.59; *p <* 0.001 on Day 2), and ISTH score (*R =* 0.38; *p =* 0.001 on Day 1 and *R =* 0.44; *p <* 0.001 on Day 2) positively correlated with the duration of hospital stay.

Serum angiopoietin-2 and sFlt-1 concentrations positively correlated with D-dimer and ISTH score (Table 2). Additionally, angiopoietin-2 significantly correlated with APTT and fibrinogen on Day 1 as well as with platelets and prothrombin time on Day 2, while sFlt-1 significantly correlated with prothrombin time on Day 2 (Table 2). The correlations between angiopoietin-2 concentrations and D-dimer were independent of C-reactive protein concentrations (β = 0.29 ± 0.13; *p =* 0.029 on Day 1, and β = 0.32 ± 0.09; *p =* 0.001 on Day 2). In addition, sFlt-1 correlated with D-dimer independently of C-reactive protein (β = 0.27 ± 0.12; *p =* 0.029 on Day 1 and β = 0.25 ± 0.12; *p =* 0.042 on Day 2).

**Figure 4:**
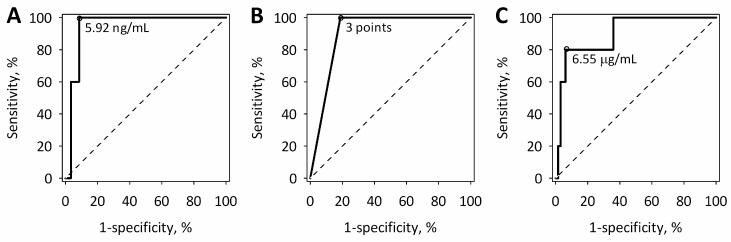
ROC curves for: angiopoietin-2 (**A**); ISTH score for overt DIC (**B**); and D-dimer (**C**) assessed on admission (within 24 h from the onset of AP symptoms) showing diagnostic utility to predict SAP. The selected cut-off values are shown on the graphs. The diagonal dashed lines are the lines of no-discrimination.

On admission, the results of all the studied tests of hemostasis and both the endothelial markers enabled the prognosis of SAP, although the diagnostic utility differed between the tests (Table 3). Among the routine tests of hemostasis, D-dimer revealed the best diagnostic utility for the prediction of severity of AP (Table 3). However, the highest values of area under the receiver operating characteristic (ROC) curves were achieved for angiopoietin-2 as a predictor of SAP, and for sFlt-1 as a predictor of MSAP+SAP (Table 3). At 24 h from the onset of AP symptoms, angiopoietin-2 at a cut-off value of 5.92 ng/mL predicted SAP with a diagnostic sensitivity of 100% and specificity of 92%; ISTH score at a cut-off value of 3 points predicted SAP with a diagnostic sensitivity of 100% and specificity of 81%; and D-dimer at a cut-off value of 6.55 µg/mL predicted SAP with a diagnostic specificity of 80% and sensitivity of 94% (Figure 4).

## 3. Discussion

In the present study, we have shown that AP is associated with abnormalities of coagulation leading to abnormal results of routinely used tests of hemostasis. Of importance, we have excluded patients with preexisting abnormalities of coagulation and none of our patients were treated with anticoagulants before or during the study. Low platelet counts, prolonged prothrombin times, prolonged APTT, and increased D-dimer concentrations observed in our patients suggest consumptive coagulopathy. Six patients (7% of the group) fulfilled the ISTH criteria for overt DIC [24]. These abnormalities correlated with both inflammation (as reflected by C-reactive protein concentrations) and endothelial dysfunction (as reflected by the concentrations of angiopoietin-2 and sFlt-1). Abnormal results of coagulation tests on admission were significantly associated with severity of AP, in particular, high plasma concentrations of D-dimer as well as ISTH score for DIC predicted more severe AP (SAP or MSAP+SAP) with reliable diagnostic accuracy.

These observations are consistent with the results of former studies. In 2006, Maeda et al. [16] reported significant associations between the results of laboratory tests of hemostasis (including platelet counts, antithrombin activity, and the concentrations of D-dimer, fibrin/fibrinogen degradation products E, and thrombin–antithrombin complexes) and severity of AP assessed in 5-stage Japanese staging system for AP. In that study, low platelet count, low antithrombin activity, and high concentrations of thrombin–antithrombin complexes and fibrin formation markers (fibrin/fibrinogen degradation product E and D-dimers) predicted death from AP with reliable diagnostic accuracy (the areas under the ROC curves between 0.768 for thrombin–antithrombin complexes and 0.926 for antithrombin activity) [16]. In addition, Radenković et al. [20] reported high diagnostic accuracy (areas under the ROC curves of 0.908 and 0.915, respectively) for D-dimer measured at admission and 24 h thereafter in the prognosis of organ failure complicating AP (pulmonary, renal failure or shock). More recently, Ke et al. [22] studied a prospective cohort of 173 patients with AP of whom 47 were diagnosed with critical AP (persistent organ failure plus infected pancreatic necrosis). D-dimer was found to be a significant predictor of critical AP (area under the ROC curve was 0.810), in contrast to C-reactive protein. Several smaller studies reported D-dimer to be a marker of severe complications, i.e., multiorgan failure or pancreatic infection or death in the course of AP [21,27,28]. On the other hand, D-dimer was also reported to significantly predict MSAP diagnosed in accordance with revised 2012 Atlanta classification [29]. Consequently, a recent position paper following the international meeting of the American Pancreatic Association and the Japanese Pancreas Society includes D-dimer in a proposed AP activity index [4]. However, from practical point of view, the lack of standardization of D-dimer measurements leads to difficulties in the interpretation of results [30]. This is reflected by discrepant cut-off values reported in the studies cited above, from 0.4 to about 1.2 µg/mL in most studies [20,21,22,27,28] and even as high as 6.1 µg/mL in the study of Maeda et al. [16]. In fact, our results confirm the data of Maeda et al. [16].

Of interest, in our study, the results of functional coagulation tests, i.e., prothrombin time, APTT and fibrinogen were associated with AP severity on admission and the associations became weaker or non-significant a day thereafter, while the highly significant association between D-dimer and AP severity persisted at 48 h from the onset of AP. One explanation may be that the functional tests are more influenced by the treatment of AP. In in the early phase of AP, intensive fluid resuscitation is introduced [2,4]. On the other hand, all patients in our study exhibited at least one abnormal result of coagulation tests, and the severity of coagulopathy as detected by the laboratory tests progressed from 24 to 48 h of AP. Thus, it seems that, in patients with more severe AP, coagulopathy develops earlier and is detectable already on admission while those with mild AP also develop some coagulation abnormalities but they became evident later.

The diagnosis of DIC is not always straightforward. Several scores are used that include the results of routine and, in some cases, also of less accessible laboratory tests [31]. In our study, we decided to use ISTH score for overt DIC, the one based on the results of routine and easily available tests [24]. Organ destruction such as pancreatitis is listed among the conditions associated with DIC, allowing the use of the clinical/laboratory scores for the diagnosis of DIC [24,31]. Indeed, several authors reported clinically significant DIC as a complication of AP [16,18,32]. However, other coagulopathies were also observed in the course of AP, such a localized thrombosis and pulmonary embolism [33,34,35,36,37] or thrombotic thrombocytopenic purpura [17,38]. The diagnosis of DIC in AP patients is further complicated by the fact that acute inflammation is a known factor leading to an increase in platelet counts [39], fibrinogen, and D-dimer concentrations [30]. Thus, in the early phase of AP, platelet counts and fibrinogen concentrations may increase due to inflammation and decrease due to consumptive coagulopathy; single laboratory result would be influenced by both processes. This is reflected by increased, rather than deceased fibrinogen observed in our cohort, including patients with SAP. As a positive acute phase protein, fibrinogen is highly influenced by inflammation, being the least sensitive marker of DIC in acute inflammatory conditions.

The pathomechanisms of activation of coagulation in AP are complex. Microvascular involvement is an important part of early pathogenesis of AP. SAP is characterized by significantly decreased blood flow in the capillaries of the pancreas [40,41], however, decreased blood flow velocity, increased vascular permeability and microvascular thrombosis are also observed in distant organs, including lungs, kidney and liver [42,43]. In 1970s, the release of proteolytic enzymes from damaged pancreas has been suggested as a causative factor for coagulopathy in AP [44]. At present, it is rather thought, that the activation or dysfunction of vascular endothelium in consequence of acute inflammation leads to procoagulant changes, including (but not confined to) the expression of tissue factor on endothelial cells, constituting the main trigger for activation of coagulation (extensively reviewed in [23]). Although indirectly, our results seem to support this assumption: in our cohort, significant correlations were found between D-dimer and inflammatory marker (C-reactive protein) as well as the markers of endothelial dysfunction (angiopoietin-2 and sFlt-1), but not between D-dimer and amylase activity. Of note, the associations between inflammation, coagulation and endothelial dysfunction are complex and reciprocal. The results of experimental studies in AP raise hope that these detrimental interactions may be in part inhibited with anticoagulant treatment [23,45,46,47,48].

The present report supplements our earlier reports regarding angiopoietin-2 [7] and sFlt-1 [8] as the markers of severity in AP; hereby we show (pathophysiologically relevant) associations of these endothelial markers with coagulopathy in AP. There is a body of evidence showing that high concentrations of angiopoietin-2 predicts more severe AP [5,6] and our results are consistent with these reports. To the contrary, according to our knowledge, our reports are the only ones showing the association between high sFlt-1 concentrations and AP severity. In 2011, Espinosa et al. [49] evaluated the relationships between soluble angiogenesis-related markers including soluble vascular endothelial growth factor receptor-1 (or sFlt-1) and AP severity and did not found the significant association. However, the discrepancy between the results of Espinosa et al. and ours may be explained by the different time-points of sample collection (Espinosa et al. took the blood samples considerably later after onset of AP symptoms than we did) and by the differences in laboratory methods. On the other hand, sFlt-1 proved useful in prediction of the severity of sepsis [9]. Still, further studies are definitely necessary to evaluate the usefulness of sFlt-1 in prediction of AP severity; such studies are especially awaited as the availability of an automated sFlt-1 assay would make it easier to introduce the marker into clinical practice.

Several limitations of our study must be admitted. First, low number of patients with SAP makes it impossible to draw definitive conclusions regarding diagnostic utility of studied tests to predict SAP. These results must be treated as hypothesis-generating and needs further evaluation in larger sample. Second, we have not measured other markers of endothelial activation or dysfunction. The degranulation of Weibel-Palade bodies of activated endothelial cells leads to the increase in plasma/serum concentrations of numerous bioactive substances besides angiopoietin-2, e.g., von Willebrand factor, interleukin-8, endothelin-1, or P-selectin [23]. The associations we have observed among angiopoietin-2, sFlt-1, coagulation abnormalities and more severe course of AP might, in fact, be mediated by other bioactive compounds. Still, this fact does not exclude the possibility for the use of the studied markers in clinical practice. Third, although we have investigated and presented the most important demographic and clinical characteristics of patients, including data on etiology of AP, and comorbidities, there are numerous unmeasured confounders that may influence our results, including diet, lifestyle and genetic factors that may influence the development of pancreatitis [50], as well as the inflammatory response [51] and the severity of coagulopathy [52].

In conclusion, our study shows that the abnormalities of coagulation are present already in the very early phase of AP (first 24 h from the onset of symptoms). The coagulopathy is associated with the severity of inflammation and endothelial dysfunction. Thus, the results of routine tests of hemostasis, and in particular, the measurement of D-dimer, may assist the prognosis of AP severity and should be taken into account in clinical practice. Although our results regarding the diagnostic utility of studied tests for the diagnosis of SAP must be treated with caution due to low number of patients with SAP, our data support the promising reports on angiopoietin-2 as an early marker of SAP. In contrast, concentrations of sFlt-1 in early phase of AP seem to predict both transient and persistent organ failure, as reflected by reasonable diagnostic accuracy for the diagnosis of MSAP plus SAP. There is a need for novel markers with high diagnostic accuracy to predict severity of AP, and the studied markers of vascular leakage and endothelial dysfunction seem promising in this setting, however, especially in case of sFlt-1, more studies are needed to verify our results. In the meantime, our results underscore the value of laboratory assessment of coagulation in clinical practice: coagulopathy detected by routine tests may be viewed as a surrogate marker of endothelial dysfunction among patients with AP.

## 4. Materials and Methods

### 4.1. Study Protocol

The prospective observational study recruited a cohort of consecutive adult patients admitted with the diagnosis of AP and treated in the Surgery Department of the District Hospital in Sucha Beskidzka, Poland. AP was diagnosed in concordance with 2012 revision of the Atlanta Classification [3], i.e., when two of the three following criteria were fulfilled: abdominal pain consistent with AP, serum amylase activity above three times greater than the upper reference limit, and characteristic findings of AP on abdominal imaging (contrast-enhanced computer tomography, magnetic resonance imaging or transabdominal ultrasonography). In addition, the inclusion criteria were admission to hospital within first 24 h from the onset of pain due to AP, and the written informed consent for the study. Patients treated with anticoagulants due to pre-existing conditions, those with pre-existing coagulopathies, as well as those with chronic pancreatitis, neoplasms, and chronic liver diseases (cirrhosis or viral hepatitis) were excluded.

The blood samples for laboratory tests were collected twice: on admission, i.e., within first 24 h from the onset of pain, and on Day 2 of hospital stay, i.e., at 48 h from the onset of pain as a symptom of AP.

DIC was diagnosed according to ISTH score for overt DIC [24]. D-dimer was used as a marker of fibrin formation. Moderately increased D-dimer concentrations were defined as 0.5–5 µg/mL (i.e., elevated up to 10-times above the reference limit), and severely increased as above 5 µg/mL.

The severity of AP was defined following 2012 revision of the Atlanta Classification [3]. MAP was diagnosed when no organ failure, local or systemic complications occurred. MSAP was diagnosed when a patient presented transient organ failure (resolving within 48 h), local (necrosis, acute necrotic collection, walled-off pancreatic necrosis) or systemic complications (exacerbation of preexisting conditions). SAP was diagnosed in patients with persistent (lasting longer than 48 h) organ failure. The diagnosis of MAP, MSAP or SAP was based on the clinical evolution of AP during the hospital stay of a patient. BISAP [25] and Glasgow [26] severity scores were calculated based on the assessment of patients during first 24 h of AP.

Additional group of healthy volunteers were recruited in order to obtain reference values for the non-routine laboratory tests, i.e., angiopoietin-2 and sFlt-1 in serum. The volunteers were men and women without any known pancreatic, renal, or liver diseases, malignancies or autoimmune diseases, without pregnancy in women, without thrombotic disorders in anamnesis, and with serum C-reactive protein concentrations below 3 mg/L. They provided written informed consent for the study. Single venous blood samples were collected from healthy volunteers into serum tubes.

The study protocol was approved by the Bioethics Committee of the Jagiellonian University (approval no. KBET/247/B/2013, permission date 28 November 2013, and 122.6120.242.2015, permission date 22 November 2015).

### 4.2. Laboratory Tests

Plasma was prepared by centrifugation of sodium citrate–anticoagulated venous blood (1 volume of citrate per 9 volumes of blood), within 20 min from blood collection. The concentrations of D-dimer, fibrinogen, as well as prothrombin time and APTT were measured on the day of blood collection, using Coatron A4 automated hemostasis analyzer (TECO Medical Instruments, Neufahrn, Germany). The reference ranges for the tests, as provided by the laboratory, were as following: <0.5 µg/mL for D-dimer, 2–4 g/L for fibrinogen, 11.4–15.5 s for prothrombin time and 26–39 s for APTT.

EDTA–anticoagulated full venous blood was used to perform complete blood counts, including platelet counts. Blood counts were performed on the day of blood collection, with the use of Sysmex XE 2100 analyzer (Sysmex Corporation, Kobe, Japan). The laboratory reference range for platelet count was 150 × 10^3^/µL–350 × 10^3^/µL.

The concentrations of C-reactive protein, glucose, creatinine, urea, bilirubin, albumin, calcium, and amylase activity were measured in serum on the day of blood collection, using automated analyzer Cobas 6000 (Roche Diagnostics, Basel, Switzerland).

All the above tests were performed in the Department of Laboratory Diagnostics, District Hospital in Sucha Beskidzka, Poland.

Serum for angiopoietin-2 and sFlt-1 measurements was obtained by centrifugation of venous blood. The blood samples collected from AP patients and from healthy volunteers were proceeded in the same way. Serum samples were aliquoted and kept frozen at −70 °C until assayed (no longer than three months). Angiopoietin-2 was measured with the Quantikine ELISA Human Angiopoietin-2 immunoassay (R&D Systems, McKinley Place, MN, USA) in the Department of Diagnostics, Chair of Clinical Biochemistry, Jagiellonian University Medical College, Kraków, Poland. The concentrations of sFlt-1 were measured by electrochemiluminescence immunoassay using Cobas 8000 analyzer (Roche Diagnostics, Mannheim, Germany) in the Department of Diagnostics, University Hospital, Kraków, Poland.

### 4.3. Statistical Analysis

Data were reported as numbers (percentage) for categories, median (lower-upper quartile) for non-normally distributed quantitative variables, and mean ± standard deviation for normally distributed quantitative variables (as tested with Shapiro–Wilk test). Chi-squared test, Mann–Whitney test, and unpaired *t*-test were used to study differences between groups, respectively. Logistic regression was used to check whether the differences between patients and controls were independent of age. Right-skewed variables were log-transformed before calculating Pearson’s correlation coefficients and before including in multiple linear regression. ROC curves were used to assess diagnostic utility of studied tests, and the results are expressed as area under the ROC curve ± standard error. Results were considered significant at p-value below 0.05. The calculations were made with the use of Statistica 12 software package (StatSoft, Tulsa, OK, USA).

## Figures and Tables

**Table 1:** Clinical characteristics of patients and the results of laboratory tests performed at 24 h from the onset of acute pancreatitis (AP) symptoms.

Variables	Patients without DIC (*N =* 63)	Patients with DIC (*N =* 6)	*p*
Age, years	59 ± 18	71 ± 16	0.1
Male sex, *N* (%)	31 (49)	4 (67)	0.4
Body mass index >30 kg/m^2^	2 (3)	0	0.7
Etiology:			
Gallstone, *N* (%)	32 (51)	3 (50)	0.9
Alcohol, *N* (%)	17 (27)	1 (17)	
Hypertriglyceridemia, *N* (%)	6 (10)	1 (17)	
Other/idiopathic, *N* (%)	8 (13)	1 (17)	
Severity:			
MAP, *N* (%)	48 (76)	1 (17)	0.003
MSAP, *N* (%)	12 (19)	3 (50)	
SAP, *N* (%)	3 (5)	2 (33)	
Pre-existing comorbidities, *N* (%)	48 (76)	6 (100)	0.2
Hypertension, *N* (%)	21 (33)	2 (33)	1.0
Diabetes, *N* (%)	9 (14)	1 (17)	0.9
Ischemic heart disease, *N* (%)	15 (24)	3 (50)	0.2
Lung diseases, *N* (%)	6 (10)	2 (33)	0.08
BISAP score, points	1 (0–1)	2 (1–2)	0.019
Glasgow severity score, points	0 (0–1)	2 (1–3)	0.035
SIRS, *N* (%)	8 (13)	1 (17)	0.8
Length of hospital stay, days	6 (5–9)	10 (8–31)	0.013
Early/late death, *N* (%)	0/1 (2)	0/2 (33)	0.018
C-reactive protein, mg/L	13.6 (2.6–86.7)	36.3 (13.7–84.5)	0.3
Leukocyte count, ×10^3^/µL	11.4 (9.6–15.4)	10.8 (9.8–11.2)	0.3
Hematocrit, %	42 ± 5	42 ± 5	1.0
Amylase, U/L	1076 (588–1844)	976 (925–1917)	0.9
Albumin, g/L	40 (37–43)	41 (34–42)	0.7
Calcium, mmol/L	2.35 (2.20–2.43)	2.29 (2.18–2.38)	0.8
Glucose, mmol/L	7.76 (6.43–10.29)	10.15 (7.79–12.25)	0.2
Creatinine, µmol/L	74.2 (63.4–97.6)	101.7 (76.0–113.4)	0.1
Urea, mmol/L	5.94 (4.26–7.08)	8.04 (6.72–12.73)	0.036
Bilirubin, µmol/L	36.0 (21.1–71.4)	36.8 (32.5–59.9)	0.9
Platelet count, ×10^3^/µL	229 ± 60	227 ± 102	0.9
Prothrombin time, s	14.4 ± 1.6	16.1 ± 1.2	0.015
APTT, s	29.5 ± 4.8	29.9 ± 6.1	0.8
Fibrinogen, g/L	2.78 (2.15–3.58)	3.18 (2.75–4.46)	0.4
D-dimer, µg/mL	1.61 (0.98–3.09)	3.99 (2.10–13.89)	0.019
Angiopoietin-2, ng/mL	3.06 (2.05–4.29)	4.97 (3.59–5.92)	0.037
sFlt-1, pg/mL	182 (163–221)	139 (110–179)	0.041

Abbreviations: DIC, diffuse intravascular coagulation; *N*, number of patients; MAP, mild acute pancreatitis; MSAP, moderately severe acute pancreatitis; SAP, severe acute pancreatitis; BISAP, bedside index of severity in acute pancreatitis; SIRS, systemic inflammatory response syndrome; APTT, activated partial thromboplastin time; sFlt-1, soluble fms-like tyrosine kinase 1.

**Table 2:** Correlations between angiopoietin-2 and sFlt-1 concentrations (log-transformed) and the results of the tests of hemostasis at 24 and 48 h from the onset of AP symptoms.

Variables	24 h				48 h			
	log (Ang-2)		log (sFlt-1)		log (Ang-2)		log (sFlt-1)	
	*R*	*p*	*R*	*p*	*R*	*p*	*R*	*p*
Platelet count	−0.18	0.2	−0.16	0.3	−0.30	0.015	−0.21	0.1
Prothrombin time	0.22	0.08	0.06	0.7	0.25	0.042	0.38	0.003
APTT	0.53	<0.001	0.19	0.2	−0.01	0.9	0.23	0.06
log (fibrinogen)	0.33	0.008	0.09	0.5	0.18	0.2	−0.04	0.8
log (D-dimer)	0.45	<0.001	0.40	<0.001	0.48	<0.001	0.27	0.028
ISTH score for overt DIC	0.33	0.008	0.40	<0.001	0.37	0.003	0.50	<0.001

Abbreviations: Ang-2, angiopoietin-2; sFlt-1, soluble fms-like tyrosine kinase 1; APTT, activated partial thromboplastin time; DIC, diffuse intravascular coagulation; ISTH, International Society on Thrombosis and Haemostasis.

**Table 3:** The values of area under the ROC curve for the studied laboratory tests of hemostasis and markers of endothelial dysfunction measured on admission (within 24 h from the onset of AP symptoms) to predict the severity of AP (diagnosis of SAP or MSAP+SAP versus less severe AP). The data for C-reactive protein are shown for comparison.

Diagnostic Tests	SAP	MSAP+SAP
Platelet count	0.803 ± 0.126	NS
Prothrombin time	0.830 ± 0.068	NS
APTT	0.768 ± 0.126	NS
Fibrinogen	0.788 ± 0.105	NS
D-dimer	0.902 ± 0.062	0.753 ± 0.070
ISTH score for overt DIC	0.913 ± 0.037	0.702 ± 0.077
Angiopoietin-2	0.946 ± 0.029	0.721 ± 0.077
sFlt-1	0.744 ± 0.064	0.810 ± 0.059
C-reactive protein	0.886 ± 0.064	0.733 ± 0.066

NS, no significant difference between the area under the ROC curve calculated for the particular test and the value of 0.5. Abbreviations: AP, acute pancreatitis; SAP, severe acute pancreatitis; MSAP, moderately severe acute pancreatitis; ROC, receiver operating characteristic; APTT, activated partial thromboplastin time; ISTH, International Society on Thrombosis and Haemostasis; DIC, diffuse intravascular coagulation; sFlt-1, soluble fms-like tyrosine kinase 1; BISAP, bedside index of severity in acute pancreatitis.
